# Study of the immunogenicity of the VP2 protein of canine parvovirus produced using an improved Baculovirus expression system

**DOI:** 10.1186/s12917-020-02422-3

**Published:** 2020-06-18

**Authors:** Dao Chang, Yangkun Liu, Yangyang Chen, Xiaomin Hu, Andrey Burov, Alexey Puzyr, Vladimir Bondar, Lunguang Yao

**Affiliations:** 1 Henan Provincal Engineering and Technology Center of Health Products for Livestock and Poultry; Key Laboratory of Ecological Security and Collaborative Innovation Centre of Water Security for Water Source Region of Mid-line of South-to-North Diversion Project of Henan Province, School of Agricultural Engineering, Nanyang Normal University, Nanyang, 473061 China; 2 Institute of Biophysics, Siberian Branch of Russian Academy of Science, Federal Research Center “Krasnoyarsk Science Center SB RAS”, 660036 Krasnoyarsk, Russia

**Keywords:** Canine parvovirus, VP2 protein, Baculovirus expression system

## Abstract

**Background:**

Canine parvovirus (CPV) is now recognized as a serious threat to the dog breeding industry worldwide. Currently used CPV vaccines all have their specific drawbacks, prompting a search for alternative safe and effective vaccination strategies such as subunit vaccine. VP2 protein is the major antigen targeted for developing CPV subunit vaccine, however, its production in baculovirus expression system remains challenging due to the insufficient yield. Therefore, our study aims to increase the VP2 protein production by using an improved baculovirus expression system and to evaluate the immunogenicity of the purified VP2 protein in mice.

**Results:**

The results showed that high-level expression of the full length VP2 protein was achieved using our modified baculovirus expression system. The recombinant virus carrying two copies of VP2 gene showed the highest expression level, with a productivity of 186 mg/L, which is about 1.4–1.6 fold that of the recombinant viruses carrying only one copy. The purified protein reacted with Mouse anti-His tag monoclonal antibody and Rabbit anti-VP2 polyclonal antibody. BALB/c mice were intramuscularly immunized with purified VP2 protein twice at 2 week intervals. After vaccination, VP2 protein could induce the mice produce high level of hemagglutination inhibition antibodies.

**Conclusions:**

Full length CPV VP2 protein was expressed at high level and purified efficiently. Moreover, it stimulated mice to produce high level of antibodies with hemmaglutination inhibition properties. The VP2 protein expressed in this study could be used as a putative economic and efficient subunit vaccine against CPV infection.

## Background

The canine parvovirus disease is an acute and highly contagious viral disease caused by canine parvovirus (CPV), which is manifested as hemorrhagic enteritis in dogs of all ages and fatal myocarditis in young puppies aged 2–3 week [1, 2]. CPV is of significant economical importance as it can cause huge losses in breeding farms [3]. Vaccination is considered as the most effective method to prevent and control CPV infection. Commercially available vaccines against CPV are mainly inactivated and live-attenuated type, however, large scale production of them is usually expensive and laborious [4, 5]. Inactivated CPV vaccines are less effective when compared with live-attenuated vaccines, thus they are not recommended for routine use [5]. Live- attenuated CPV vaccines are effective and widely used, but a series of CPV-2-like strains were identified from sick and vaccinated dogs and deduced to evolve from live-attenuated vaccine strains [6]. To overcome these problems, attempts were made to develop alternative vaccines, such as subunit vaccine.

Canine parvovirus is a small, non-enveloped virus containing linear ssDNA (single-stranded DNA) genomes of approximately 5 kb, which encodes three structural proteins (VP1, VP2 and VP3) and two non-structural proteins (NS1 and NS2) [7]. The VP2 protein is the predominant structural protein of CPV which constitute about 90% of viral capsid and plays an important role in the transmission and infection of CPV [8, 9]. In addition, VP2 protein contains several important B cell epitopes in the N-domain and loop-domain, which could induce effective neutralizing antibody during the infection of CPV [8]. Therefore, the VP2 protein is generally considered as a potent protective antigen and a promising candidate for the CPV subunit vaccine.

The baculovirus expression vector system (BEVS) is an excellent eukaryotic expression system with advantages of the high-level expression of foreign proteins and the ability of post-translational modification, thus it has been widely used in the production of recombinant protein and subunit vaccine [10, 11]. An improved baculovirus express system based on MultiBac system was constructed in a previous study [12, 13]. The recombinant baculovirus carrying multiple expression cassettes could be produced rapidly and simply by using Bacmid-containing diminopimelate-auxotrophic *Escherichia coli* infecting insect cells [13]. In this study, the improved baculovirus expression system was used to produce VP2 protein by co-expression two copies of VP2 gene, and the immunogenicity of the purified protein in mice was investigated.

## Results

### Identification of the recombinant bacteria containing positive bacmids

**Fig. 1:**
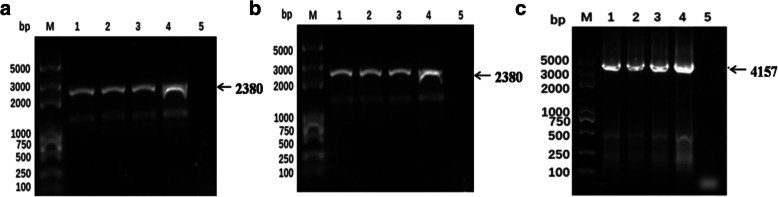
PCR analysis of the recombinant bacmids, using forward primers of VP2 gene and M13 reverse primers. Lane M: Trans2K Plus DNA Marker; (**a**) 1–3: PCR product of AcMultiBac-IM-p10-VP2; (**a**) 4: PCR product of plasmid pYBDM-IM-p10-VP2; (**b**) 1–3: PCR product of AcMultiBac-IM-ph-VP2; (**b**) 4: PCR product of plasmid pYBDM-IM-ph-VP2; (**c**) 1–3: PCR product of AcMultiBac-IM-2PV2; (**c**) 4: PCR product of plasmid pYBDM-IM-ph-VP2 + p10-VP2; (**a-c**) 5: PCR product of AcMultiBacmid/rSW106/asd^−^/inv^+^

After transferring the recombinant donor plasmids into competent *E. coli* AcMultiBacmid/rSW106/asd^−^/inv^+^ cells, recombinant bacmids were obtained and the correct insertion of the target genes was confirmed by PCR analysis. Recombinants were amplified with one gene-specific primer and one M13 primer. If transposition has occurred, the recombinant baculovirus could amplify a 2380 bp band (including the size of VP2 gene 1777 bp and primers 603 bp) or 4157 bp band (including the size of two copies of VP2 gene 3554 bp and primers 603 bp) whereas non-recombinant baculovirus could not amplify any band, suggesting that the VP2 gene from the three constructs, had been successfully recombined into bacmid shuttle vectors (Fig. 1).

### Production of recombinant baculovirus

**Fig. 2:**
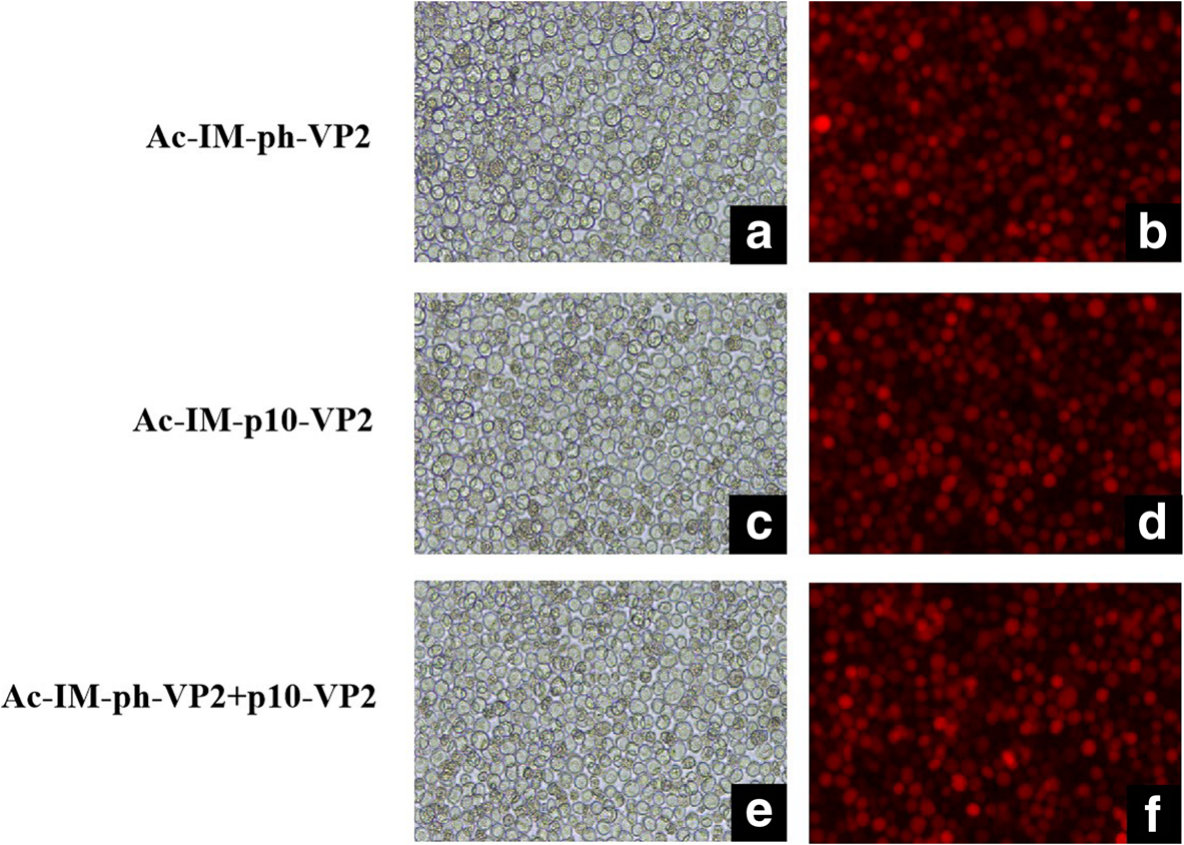
Microscopy of Sf9 cells infected with Ac-IM-ph-VP2, Ac-IM-p10-VP2 and Ac-IM-ph-VP2 + p10-VP2. The visible light image (**a, c, e**) and fluorescence image (**b, d, f**) were captured at 120 h post-infection

**Fig. 3:**
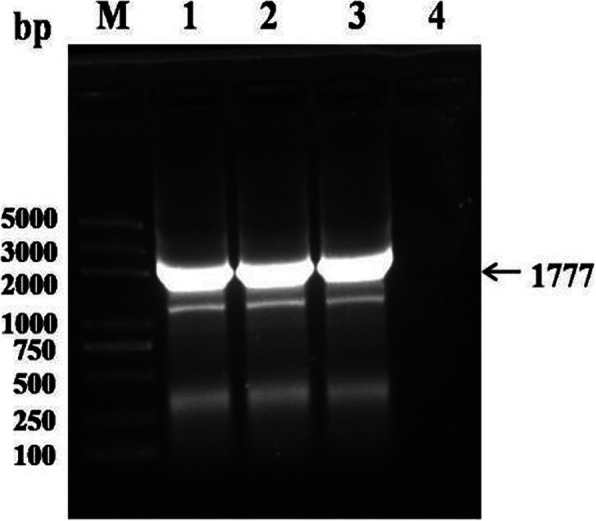
Identification of the baculoviral genomic DNA samples by PCR. Lane M: DL2000 Marker; Lane 1: genomic DNA of the recombinant virus Ac-IM-ph-VP2; Lane 2: genomic DNA of the recombinant virus Ac-IM-p10-VP2; Lane 3: genomic DNA of the recombinant virus Ac-IM-ph-VP2 + p10-VP2; Lane 4: negative control, genomic DNA of uninfected Sf9 cells

The recombinant bacteria carrying positive bacmids was used to infect Sf9 cells to produce recombinant baculovirus as described previously [13]. The Sf9 cells were infected with the recombinant baculovirus at an MOI of 0.1 for virus amplification. When examined by fluorescence microscopy, it was found that obvious red fluorescence in virus-infected Sf9 cells (Fig. 2). The presence of the VP2 gene in recombinant viruses was confirmed by direct PCR of the viral genomic DNA. As expected, PCR amplification of the recombinant viral genomic DNA revealed a specific band of 1.77 kb. Non-specific amplification was not observed in the negative control sample (Fig. 3). These results indicated that VP2 gene was inserted into recombinant baculovirus.

### Expression of recombinant VP2 protein in insect cells

**Fig. 4:**
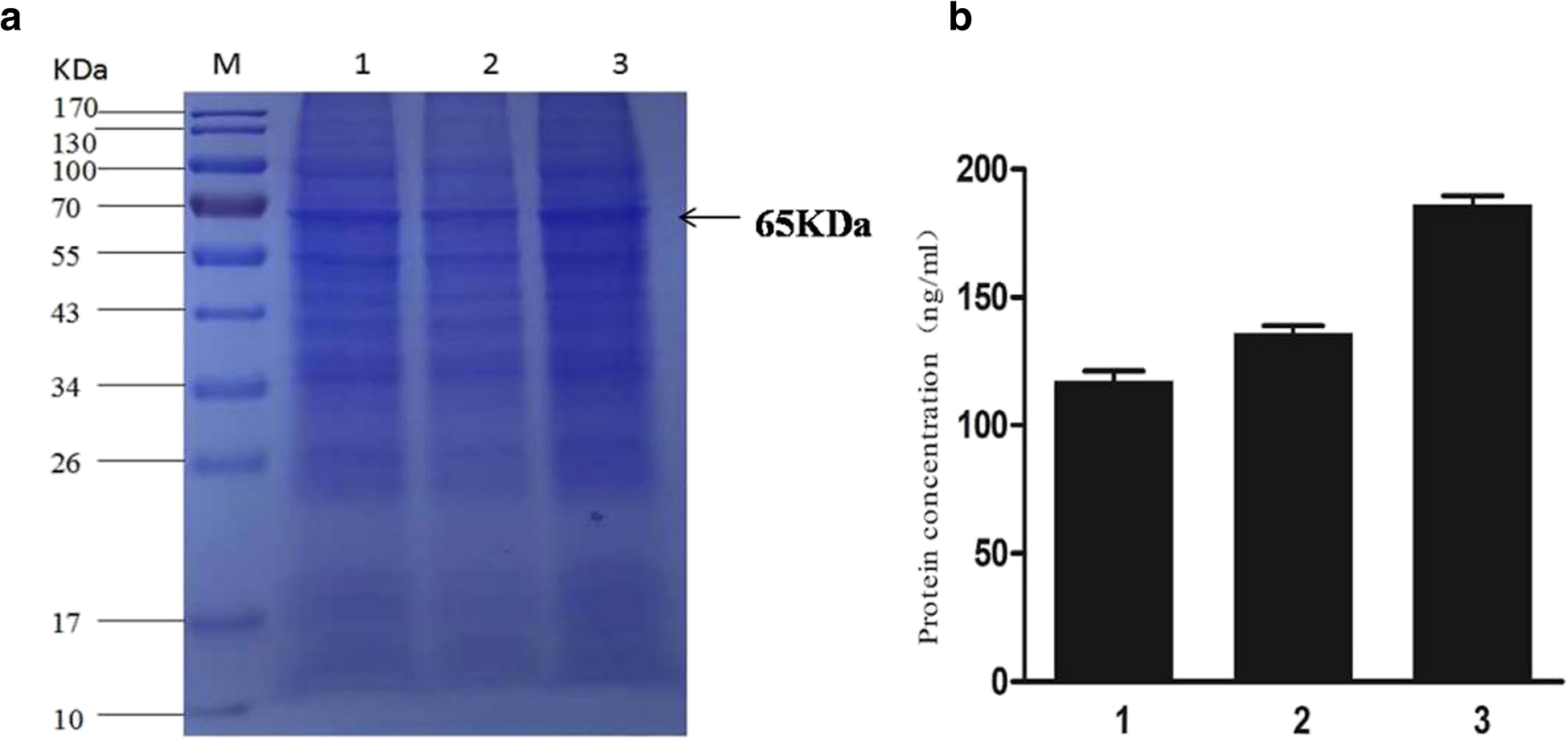
Expression of recombinant VP2 protein in insect cells. **a** SDS-PAGE analysis of VP2 expression in different virus-infected Sf9 cells at 96 h p.i. **b** The expression level of CPV VP2 protein in different virus-infected cells. 1: recombinant baculovirus Ac-IM-p10-VP2; 2: recombinant baculovirus Ac-IM-ph-VP2; 3: recombinant baculovirus Ac-IM-ph-VP2 + p10-VP2

2 × 10^8^ of Sf9 cells (100 mL cell culture) were infected respectively with the recombinant baculoviruses Ac-IM-ph-VP2, Ac-IM-p10-VP2 and Ac-IM-ph-VP2+ p10-VP2 at a MOI of 5 for protein expression. At 96hpi, the expression of VP2 protein in different virus-infected insect cells was determined by SDS-PAGE, and then the expression level of VP2 protein was quantified with the His Tag ELISA Detection Kit. As shown in Fig. 4a and Additional file 1, the recombinant VP2 band had a size of 65 kDa, which correlates to its expected size. In addition, VP2 expression is considerable higher in Sf9 cells infected with Ac-IM-ph-VP2 + p10-VP2 (0.186 mg/mL cell suspension or 186 mg/L) when compared to Ac-IM-ph-VP2 (0.139 mg/mL, or 139 mg/L) and Ac-IM-p10-VP2 (0.117 mg/mL or 117 mg/L), respectively (Fig. 4b). A high expression level of the full length VP2 protein was achieved when using our modified system. And the recombinant virus with two copies of VP2 gene showed the highest level, which is about 1.4–1.6 fold that of the virus with one copy. Therefore, Ac-IM-ph-VP2 + p10-VP2 was used as seed virus to infect Sf9 cells in the following experiments.

### Purification and characterization of recombinant VP2 protein

**Fig. 5:**
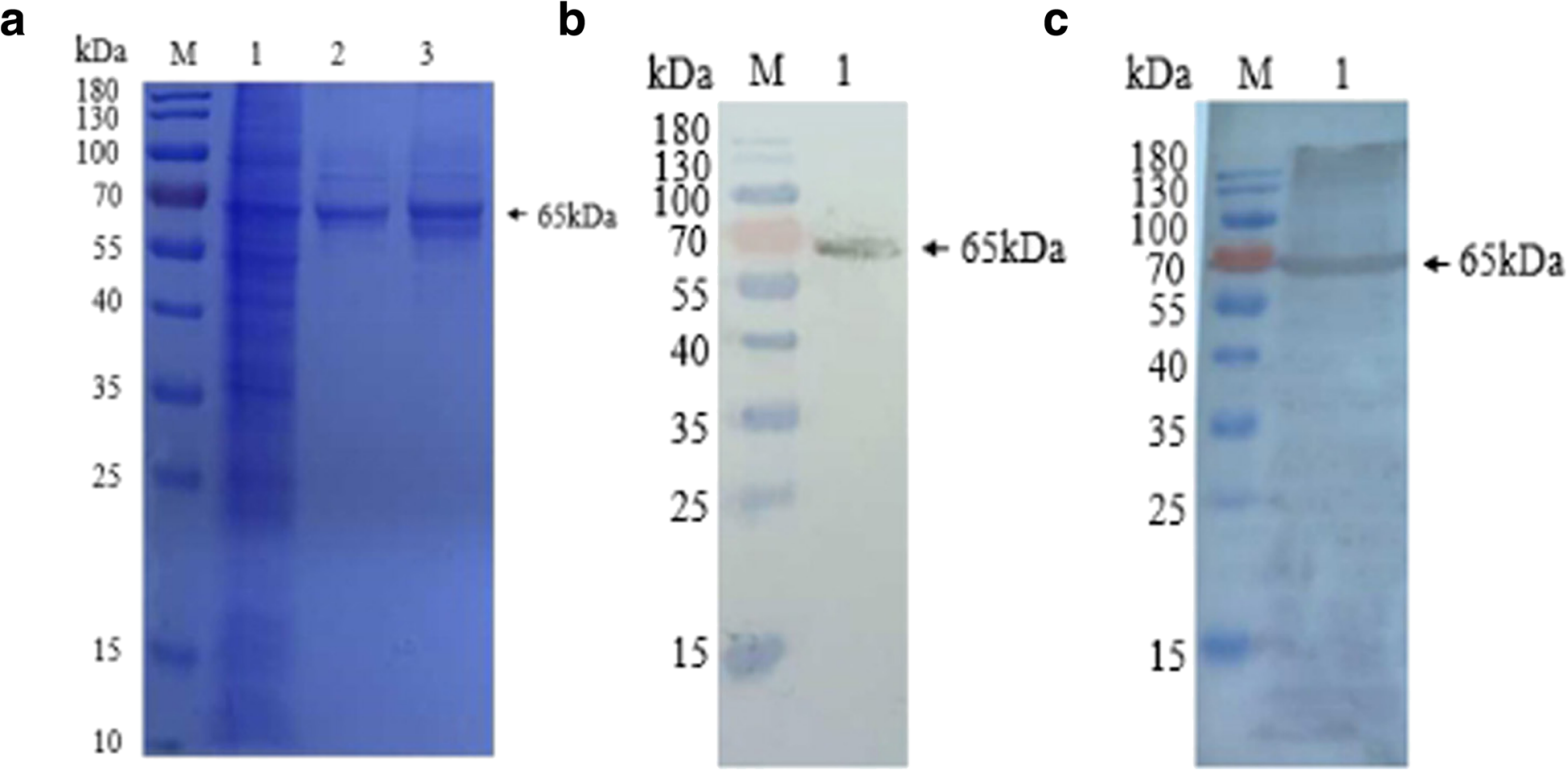
Purification and characterization of recombinant VP2 protein. **a** The SDS-PAGE analysis of purified recombinant VP2 protein. Lane M: PageRuler™ Prestained Protein Ladder, 10 to 180 kDa; Lane 1: ultrasound supernatant of the recombinant baculovirus Ac-IM-ph-VP2 + p10-VP2 infected Sf9 cells; Lane 2: 200 mM Imidazole eluent; Lane 3: 300 mM Imidazole eluent. **b** Western blot analysis of purified VP2 protein with Mouse anti-His monoclonal antibodies. Lane 1: purified VP2 protein; **c** Western blot analysis of purified VP2 protein with Rabbit anti-VP2 polyclonal antibodies. Lane 1: purified VP2 protein

Based on its C-terminal His-tag, the recombinant VP2 protein was purified by Ni^2+^- affinity chromatography. The SDS-PAGE results showed that a specific band at 65 kDa was observed in the elution buffer (Fig. 5a; Additional file 1), and the purity of recombinant VP2 protein was 90.5%. Western blot analysis further confirmed that the purified protein was in fact VP2 protein when reacted with Mouse anti-His monoclonal antibodies (Fig. 5b; Additional file 1) or Rabbit anti-VP2 polyclonal antibodies (Fig. 5c; Additional file 1).

### Serum antibodies against recombinant VP2 protein in mice

**Fig. 6:**
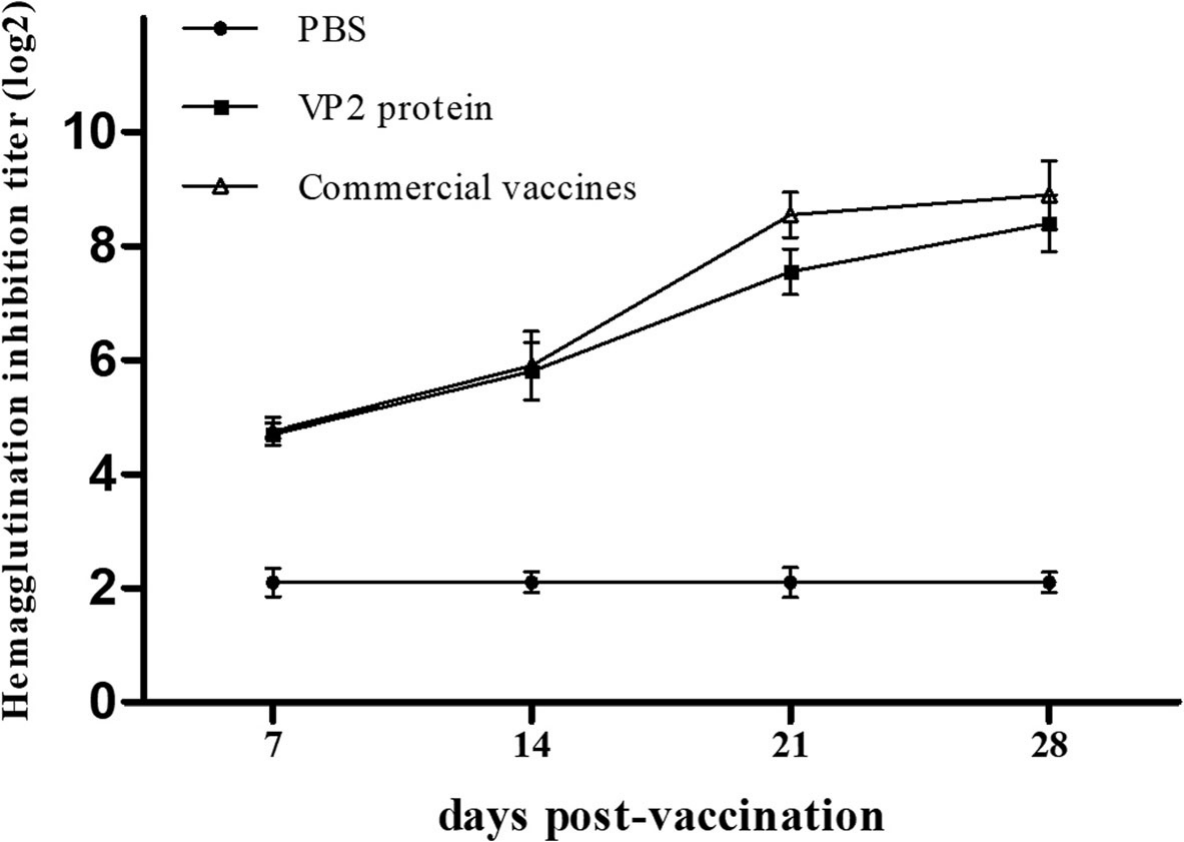
Hemagglutination inhibiting (HI) antibody titers in mice vaccinated with the VP2 protein at different days post-vaccination. Results represent mean values of each group sera samples ± SD from three independent experiments. The statistical significance of antibody titter differences between different groups were analyzed by one-way ANOVA statistical analysis and significant difference is expressed as *P* < 0.05 (*)

Serum samples collected from 5 mice of each group were subjected to the HI test to determine the antibody titer against CPV. As shown in Fig. 6, all PBS buffer-inoculated mice were negative for HI antibodies throughout the study. In group A mice that were vaccinated with VP2 protein, HI antibodies were positive at 7 dpv and steadily increased thereafter, and then HI antibody titers reached the highest (1: 2^8.4^) at 28 dpv. In group B, commercial live-attenuated vaccine induced slightly higher levels of HI antibodies than that of the VP2 protein, however, there is no significant difference was found between the commercial CPV vaccine and VP2 protein group (*P* > 0.05). These results indicated that the VP2 protein could induce a specific antibody response as the commercial live-attenuated vaccine.

## Discussion

VP2 protein of Canine parvovirus (CPV) is the major determinant which elicits specific neutralizing antibodies, and has been used as recombinant protein-based subunit vaccine in previous studies [14–16]. In addition, subunit vaccine comprise selected pathogen specific antigens are a safe alternative to traditional whole organism vaccines [17]. In this study, we expressed the full length VP2 capsid protein as a first step in the development of CPV subunit vaccine. An improved baculovirus expression system was used to increase the expression of VP2 protein. Further, the immunogenicity of purified VP2 proteins were evaluated in mice. The results showed that the VP2 proteins were expressed in Sf9 cells at a high level, and the purified recombinant VP2 proteins could induce strong immune responses in mice.

Previous studies reported that VP2 protein could be produced in *E. coli* and insect expression system [15, 16, 18–21]. Although *E. coli* expression system has been used widely for recombinant proteins production in laboratory and industrial scale due to its simplicity and economy, the solubility of target proteins could sometimes be low [22–24]. Particularly, VP2 protein of CPV, has high molecular weight and weak hydrophilicity, which make it prone to aggregate easily in inclusion bodies. Previous reports demonstrated that SUMO tag or molecular chaperone Tf16 could promote the solubility of VP2 proteins, however, the procedure is complicated and inconvenient [4, 25]. Therefore, the baculovirus insect cell expression system (BEVS) was used to produced VP2 protein in this study.

So far, Bac-to-Bac system is a common utilized BEVS for the production of heterologous proteins because it has many advantages such as proper post-translational modifications and protein processing [26]. Bac-to-Bac system enables protein expression controlled by its highly active promoters of polyhedrin (polh) or p10. Here, we report here that the employment of the improved system permits to generate recombinant baculovirus containing single or double VP2 gene inserts. After comparing the VP2 protein yield after infection with Ac-IM-p10-VP2 and Ac-IM-ph-VP2, we found similiar VP2 protein yield upon polh- and p10- promoter driven expression. In addition, simultaneous expression from both promoters together, results in slightly increased CPV VP2 production (Fig. 4b). The enhancement of VP2 expression driven by both promoters was 1.4 and 1.6-fold than single polh- and p10-promoter driven expression, respectively. These findings are consistent with previous studies which reported similar conclusion upon single and combined polh and p10 promoter driven expression of HPV57 L1 protein and insulin-like growth factor-interleukin-3 chimeras [27, 28]. However, the detailed mechanism of this increase in protein expression by combined promoter is still unclear, we speculate that it may due to multiple transcriptional start sites will increase RNA levels. Therefore, our study demonstrates that combined expression from both p10 and polh promoters together can be an effective way used to increase the expression level of heterologous proteins. In this study, the yield obtained was 186 mg/L, which is an acceptable amount of recombinant protein in terms of the possibility of producing a novel vaccine.

Currently, HI is considered as the standard method for detection of CPV antibodies [29]. To evaluate the immunogenicity of an experimental vaccine based on the purified VP2 protein, HI antibodies were obtained in mice. As shown in Fig. 6, HI antibody titers increased rapidly after the second immunization. It is remarkable that the maximum titers of HI antibody in VP2 protein immunity group were 1: 2^8.4^ at 28 dpv. Although it induced slightly lower levels of HI antibodies than that of commercial live-attenuated vaccine, no significant difference was found between the two groups (*P* > 0.05). This result suggested that CPV VP2 protein could induce almost the same effective immune response in mice as CPV live-attenuated vaccine. Therefore, our study showed pereliminary results obtained in mice, in which the VP2 protein expressed in this system could induce similar HI antibody titers to prevent CPV infection in dogs.

## Conclusion

In conclusion, our study describes the improvement in gene expression level and the high immunogenicity of CPV VP2 protein produced in insect cell lines using an improved system. These results suggest that, further studies should be done to prove if CPV VP2 protein, might be a safe, convenient and effective vaccine for preventing CPV, since the use of the improved baculovirus system to produce viral proteins, is low-cost and attractive.

## Methods

### Materials

*Spodoptera frugiperda* (Sf9) cell lines were grown and maintained at 27 °C in SF900III medium (Invitrogen Corporation, USA). Plasmid pET28a-CPV-VP2 containing CPV VP2 gene (GenBank: MK518021.1) and Rabbit anti-VP2 polyclonal antibody were kindly provided by Dr. Qinghai Tang [30]. The improved baculovirus expression system includes the vector pYBDM-IM (a mcherry fragment driven by IRES is insert into the *Sph*I and *Kpn*I sites of pFBDM), and the *Esherichia coli* AcMultiBacmid/rSW106/asd^−^/inv^+^ strain were constructed as described previously [13].

### Construction of recombinant transfer vectors

**Fig. 7:**
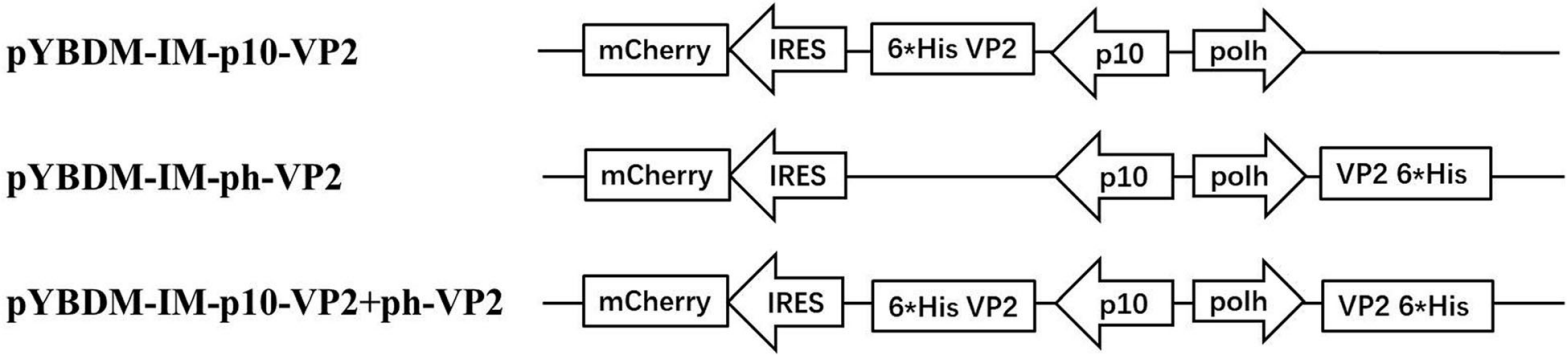
Schematic diagram of different transfer vectors containing CPV VP2 gene. Polh: polyhedrin promoter; p10: p10 promoter; IRES: internal ribosome entry site derived from *Rhopalosiphum padi* virus; mCherry: cDNA of red fluorescent protein derived from *mushroom coral*

To facilitate expression of the VP2 genes in insect cells, full-length VP2 gene was amplified by PCR from plasmid pET28a-CPV-VP2. The primers used for VP2 gene amplification were VP2 Forward primer (5′-GGATCCCGGGATGAGTGATGG AGCAGT-3′ containing one site of *Bam*HI and overlapping the *Sma*I site) and VP2 Reverse primer (5′-TCTAGAGTCGACTTAATATAATTTTCTAG-3′ containing one site of *Xba*I and one of *Sal*I). The PCR product encoding VP2 ORF was treated with *Bam*HI*/Xba*I and cloned into the multiple cloning sites (MCS) under the polyhedrin promoter, the resultant plasmid was designated pYBDM-IM-ph-VP2. Similarly, the amplified fragment was inserted into *Sma*I*/Xho*I restriction sites, downstream from the p10 promoter of the vector pYBDM-IM or pYBDM-IM-ph-VP2, to generate the plasmid of pYBDM-IM-p10-VP2 or pYBDM-IM-ph-VP2 + p10-VP2 respectively (Fig. 7). All constructs were confirmed by DNA sequencing.

### Generation of recombinant bacmids

Individual recombinant plasmids were transformed into *E. coli* AcMultiBacmid/rSW106/asd^−^/inv^+^ competent cells, allowing the transposition of CPV VP2 gene to Bacmid between the mini-Tn7 element on the transfer vector and the mini-att Tn7 target site on the Bacmid to generate the recombinant bacmids. Prepared recombinant bacmids were characterized by white-blue and PCR screening using VP2 forward primer and M13 reverse primer to confirm the insertion of the VP2 gene.

### Production of recombinant baculoviruses

*E.coli* AcMultiBacmid/rSW106/asd^−^/inv^+^ cells with recombinant VP2 gene were grown in LB broth supplemented with 0.5 mM DAP, 10 μg/mL tetracycline, 7 μg/mL gentamicin, 25 μg/mL spectinomycin and 50 μg/mL kanamycin at 30 °C. The overnight culture were collected by centrifugation (3000 g) and resuspended in distilled ultrapure water for three times. The pellet was resuspended in SF900III medium and adjusted to different densities (10^5^–10^8^ cells/mL). Sf9 cells at 10^5^/mL were incubated overnight in 24-well plates (70–80% confluent single layer). After removing the medium, 500 μL bacterial cells at different concentrations were added to each well to infect Sf9 cells [13]. After culturing at 27 °C for 4–5 h, each well was washed three times. Five hundred microliter fresh SF900III medium was then added and incubated for 3–5 days. When the mCherry fluorescence was observed using reverted fluorescence microscopy (507 nm excitation), indicating that Sf9 cells were infected successfully. The supernatant containing recombinant baculovirus was harvested and infected again with Sf9 cells. Titers of the baculovirus were determined by a plaque assay.

### Production and purification of CPV VP2 protein

2.0 × 10^8^ of Sf9 insect cells were grown in 100 mL volumes in 500 mL polycarbonate Erlenmeyer flasks and incubated in an orbital shaker incubator at 125 rpm and 28 °C. The initial cell density was 2.0 × 10^5^ cells/mL. The cultures were infected with the recombinant baculoviruses, Ac-IM-ph-VP2, Ac-IM-p10-VP2 and Ac-IM-ph-VP2 + p10-VP2 at a multiplicity of infection (MOI) of 5. At 96 h post infection (h.p.i.), the cells were harvested by centrifugation (1000 g, 10 mins, 4 °C) and lysed by sonication. The lysed Sf9 cells were centrifuged at 12, 000 g for 10 min, the supernatant was purified using Ni-NTA agarose according to the manufacturer’s instructions. Purity of the VP2 protein is calculated from the SDS-PAGE gels using ImageJ 1.46r program (National Institute of Health, USA). The concentration of VP2 protein was measured with the His Tag ELISA Detection Kit (GenScript, Nanjing, China).

### Western blot analysis of recombinant CPV VP2 protein

The expression of CPV VP2 proteins from Ac-IM-ph-VP2, Ac-IM-p10-VP2 and Ac-IM-ph-VP2 + p10-VP2 were determined by Western blot assay. The supernatant of samples, which were purified as described above, were separated by 12% SDS-PAGE and transferred onto polyvinylidene fluoride membranes and then blocked with 10% skimmed milk for 2 h. After five washes with PBST (PBS plus 0.05% Tween-20) for 5 min each time, the membranes were incubated overnight with mouse anti-His monoclonal antibody (1: 5000 dilution) and rabbit anti-VP2 polyclonal antibody (1: 200 dilution) at 4 °C, respectively. After five washes with PBST for 5 min each time, the membranes were incubated with HRP-conjugated goat anti mouse IgG antibody (1: 2000 dilution) and HRP-conjugated goat anti rabbit IgG antibody (1: 2000 dilution), respectively. After five washes with PBST for 5 min each time, detection was performed with DAB (3, 3′-diaminobenzidine) solution (Boshide, Wuhan, China).

### Animals experiment

The purified VP2 proteins from Ac-IM-ph-VP2 + p10-VP2 were adjusted at a final concentration of 1 mg/mL. All animal protocols were performed in accordance with the guidelines of the ethical committee of Nanyang Normal University. The mice were purchased from Wuhan Biological products Research Institute Co., Ltd. Fifteen BALB/c mice (6-week-old, female) were randomized into three groups (*n* = 5). Mice in group A were intramuscularly injected with 100 μL of purified VP2 protein mixed with Freund’s adjuvant. Mice in group B were intramuscularly injected with 100 μL commercial live-attenuated vaccine as a positive control. Mice in group C were intramuscularly injected with 100 μL PBS as a negative control. Mice from all groups were injected twice at 2-week intervals (Days 0 and 14). Blood samples were collected from the forelimb vein at 0, 7, 14, 21 and 28 days post-vaccination (dpv). At the end of experiment, animals were euthanized by the intravenous administration of an overdose of sodium pentobarbital.

### Hemagglutination inhibition (HI) test

Serum samples from mice were inactivated at 56 °C for 30 min, then serially diluted two-fold (25 μL serum) in V-96-well plates. Subsequently 25 μL 4 hemagglutination units of CPV NY strain (provided by Dr. Qinghai Tang [30]) were added. The mixture were incubated at 37 °C for 1 h after which 50 μL 0.8% pig erythrocytes were added. Hemagglutination inhibition antibody titers were expressed as the reciprocal of the highest serum dilution that completely inhibited hemagglutination.

### Statistical analysis

The experimental data were analyzed using a one-way analysis of variance (ANOVA), combined with Tukey’s post hoc test. *P* < 0.05 was considered statistically significant.

## Supplementary information

**Additional file 1 **Original image of **Fig. 5a**. SDS-PAGE analysis of VP2 expression in different virus-infected Sf9 cells at 96 h post infection. M: PageRuler™ Prestained Protein Ladder, 10 to 170 kDa; 1: recombinant baculovirus Ac-IM-p10-VP2; 2: recombinant baculovirus Ac-IM-ph-VP2; 3: recombinant baculovirus Ac-IM-ph-VP2 + p10-VP2. Original image of **Fig. 6a**. The SDS-PAGE analysis of purified recombinant VP2 protein. M: PageRuler™ Prestained Protein Ladder, 10 to 180 kDa; 1: ultrasound supernatant of the recombinant baculovirus Ac-IM-ph-VP2 + p10-VP2 infected Sf9 cells; 2: 200 mM Imidazole eluent; 3: 300 mM Imidazole eluent; 4: 500 mM Imidazole eluent; 5: First eluent of 500 mM Imidazole; 6: Second eluent of 500 mM Imidazole; 7: Third eluent of 500 mM Imidazole; 8: Fourth eluent of 500 mM Imidazole. Original image of **Fig. 6b**. Western blot analysis of purified VP2 protein with Mouse anti-His monoclonal antibodies. M: PageRuler™ Prestained Protein Ladder, 10 to 180 kDa; 1: First eluent of 200 mM Imidazole; 2: Second eluent of 200 mM Imidazole; 3: Third eluent of 200 mM Imidazole. Original image of **Fig. 6c**. Western blot analysis of purified VP2 protein with Rabbit anti-VP2 polyclonal antibodies. M: PageRuler™ Prestained Protein Ladder, 10 to 180 kDa; 1: First eluent of 200 mM Imidazole; 2: Second eluent of 200 mM Imidazole; 3: Third eluent of 200 mM Imidazole.

## Data Availability

The datasets used and/or analysed during the current study are available from the corresponding author on reasonable request.

## Abbreviations

BEVS: Baculovirus Expression Vector System
CPV: Canine parvovirus
DAB: 3,3′-diaminobenzidine
DAP: 2,6-diaminopimelic
dpv: Days post-vaccination
ELISA: Enzyme linked immunosorbent assay
HI: Hemagglutination inhibition
hpi: Hours post infection
HPV: Human papilloma virus
MOI: Multiplicity of infection
ORF: Open reading frames
SDS-PAGE: Sodium dodecyl sulfate-polyacrylamide gel electrophoresis
ssDNA: Single-stranded DNA
