# Supplementary material for: Study of the immunogenicity of the VP2 protein of canine parvovirus produced using an improved Baculovirus expression system
Source: BMC Vet Res. 2020 Jun 18;16:202. doi: 10.1186/s12917-020-02422-3 (PMC7301529; doi:10.1186/s12917-020-02422-3)
Supplement: Supplementary file 1 — Additional file 1 Original image of Fig. 5a. SDS-PAGE analysis of VP2 expression in different virus-infected Sf9 cells at 96 h post infection. M: PageRuler™ Prestained Protein Ladder, 10 to 170 kDa; 1: recombinant baculovirus Ac-IM-p10-VP2; 2: recombinant baculovirus Ac-IM-ph-VP2; 3: recombinant baculovirus Ac-IM-ph-VP2 + p10-VP2. Original image of Fig. 6a. The SDS-PAGE analysis of purified recombinant VP2 protein. M: PageRuler™ Prestained Protein Ladder, 10 to 180 kDa; 1: ultrasound supernatant of the recombinant baculovirus Ac-IM-ph-VP2 + p10-VP2 infected Sf9 cells; 2: 200 mM Imidazole eluent; 3: 300 mM Imidazole eluent; 4: 500 mM Imidazole eluent; 5: First eluent of 500 mM Imidazole; 6: Second eluent of 500 mM Imidazole; 7: Third eluent of 500 mM Imidazole; 8: Fourth eluent of 500 mM Imidazole. Original image of Fig. 6b. Western blot analysis of purified VP2 protein with Mouse anti-His monoclonal antibodies. M: PageRuler™ Prestained Protein Ladder, 10 to 180 kDa; 1: First eluent of 200 mM Imidazole; 2: Second eluent of 200 mM Imidazole; 3: Third eluent of 200 mM Imidazole. Original image of Fig. 6c. Western blot analysis of purified VP2 protein with Rabbit anti-VP2 polyclonal antibodies. M: PageRuler™ Prestained Protein Ladder, 10 to 180 kDa; 1: First eluent of 200 mM Imidazole; 2: Second eluent of 200 mM Imidazole; 3: Third eluent of 200 mM Imidazole. [file 12917_2020_2422_MOESM1_ESM.docx]

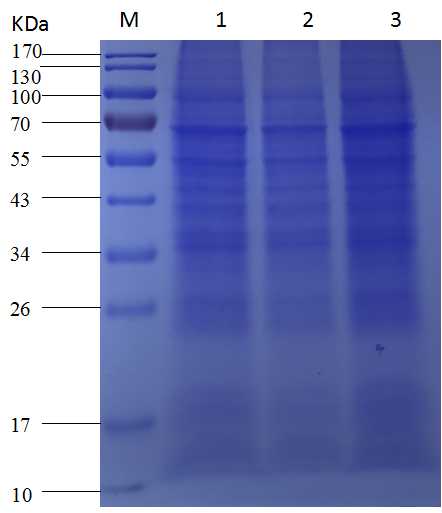


**Original image of Fig 5A. SDS-PAGE analysis of VP2 expression in different virus-infected Sf9 cells at 96 hours post infection.** M: PageRuler™ Prestained Protein Ladder, 10 to 170 kDa; 1: recombinant baculovirus Ac-IM-p10-VP2; 2: recombinant baculovirus Ac-IM-ph-VP2; 3: recombinant baculovirus Ac-IM-ph-VP2+p10-VP2.


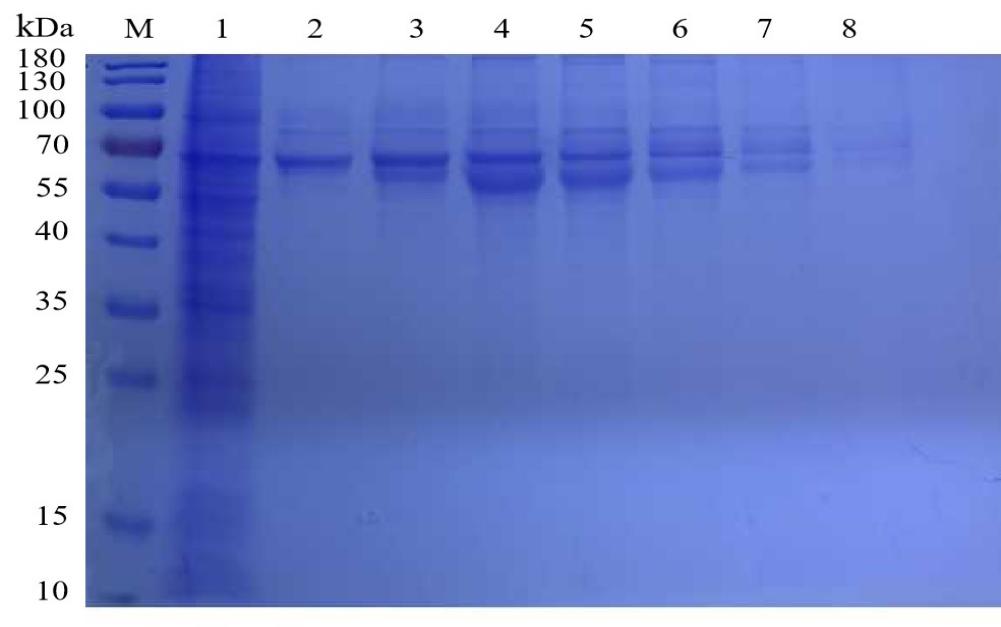


**Original image of Fig 6A. The SDS-PAGE analysis of purified recombinant VP2 protein.** M: PageRuler™ Prestained Protein Ladder, 10 to 180 kDa; 1: ultrasound supernatant of the recombinant baculovirus Ac-IM-ph-VP2+p10-VP2 infected Sf9 cells; 2: 200 mM Imidazole eluent; 3: 300 mM Imidazole eluent; 4: 500 mM Imidazole eluent; 5: First eluent of 500 mM Imidazole; 6: Second eluent of 500 mM Imidazole; 7: Third eluent of 500 mM Imidazole; 8: Fourth eluent of 500 mM Imidazole.


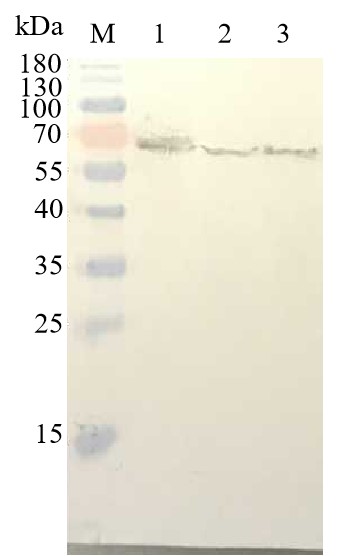


**Original image of Fig 6B. Western blot analysis of purified VP2 protein with Mouse anti-His monoclonal antibodies.** M: PageRuler™ Prestained Protein Ladder, 10 to 180 kDa; 1: First eluent of 200 mM Imidazole; 2: Second eluent of 200 mM Imidazole; 3: Third eluent of 200 mM Imidazole.


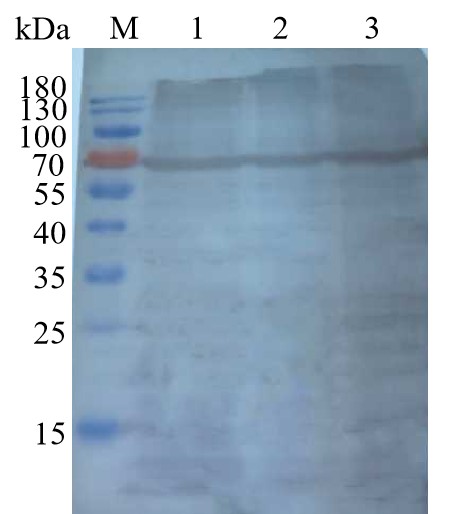


**Original image of Fig 6C. Western blot analysis of purified VP2 protein with Rabbit anti-VP2 polyclonal antibodies.** M: PageRuler™ Prestained Protein Ladder, 10 to 180 kDa; 1: First eluent of 200 mM Imidazole; 2: Second eluent of 200 mM Imidazole; 3: Third eluent of 200 mM Imidazole.
